# Physiological Analysis and Proteome Quantification of Alligator Weed Stems in Response to Potassium Deficiency Stress

**DOI:** 10.3390/ijms20010221

**Published:** 2019-01-08

**Authors:** Li-Qin Li, Cheng-Cheng Lyu, Jia-Hao Li, Zhu Tong, Yi-Fei Lu, Xi-Yao Wang, Su Ni, Shi-Min Yang, Fu-Chun Zeng, Li-Ming Lu

**Affiliations:** College of Agronomy, Sichuan Agriculture University, Chengdu 611130, China; liliqin88@163.com (L.-Q.L.); chengchengLyu@163.com (C.-C.L.); 18889589812@139.com (J.-H.L.); t17313102122@163.com (Z.T.); sarklu@126.com (Y.-F.L.); wxyrtl@163.com (X.-Y.W.); ns13@163.com (S.N.); yangshimin1@163.com (S.-M.Y.); zengfuchun78@163.com (F.-C.Z.)

**Keywords:** *Alternanthera philoxeroides*, proteomic, stem, potassium, stress

## Abstract

The macronutrient potassium is essential to plant growth, development and stress response. Alligator weed (*Alternanthera philoxeroides*) has a high tolerance to potassium deficiency (LK) stress. The stem is the primary organ responsible for transporting molecules from the underground root system to the aboveground parts of the plant. However, proteomic changes in response to LK stress are largely unknown in alligator weed stems. In this study, we investigated the physiological and proteomic changes in alligator weed stems under LK stress. First, the chlorophyll and soluble protein content and SOD and POD activity were significantly altered after 15 days of LK treatment. The quantitative proteomic analysis suggested that a total of 296 proteins were differentially abundant proteins (DAPs). The functional annotation analysis revealed that LK stress elicited complex proteomic alterations that were involved in oxidative phosphorylation, plant-pathogen interactions, glycolysis/gluconeogenesis, sugar metabolism, and transport in stems. The subcellular locations analysis suggested 104 proteins showed chloroplastic localization, 81 proteins showed cytoplasmic localization and 40 showed nuclear localization. The protein–protein interaction analysis revealed that 56 proteins were involved in the interaction network, including 9 proteins involved in the ribosome network and 9 in the oxidative phosphorylation network. Additionally, the expressed changes of 5 DAPs were similar between the proteomic quantification analysis and the PRM-MS analysis, and the expression levels of eight genes that encode DAPs were further verified using an RT-qPCR analysis. These results provide valuable information on the adaptive mechanisms in alligator weed stems under LK stress and facilitate the development of efficient strategies for genetically engineering potassium-tolerant crops.

## 1. Introduction

Potassium (K^+^) is the most important and abundant nutrient ion in living plant cells, and it plays crucial roles in many physiological and biochemical processes, such as photosynthesis, protein synthesis, enzyme activation, osmotic regulation, ion homeostasis, and stomata movement [1]. Under optimum growth conditions, the cytoplasmic K^+^ concentration in plant cells is ~100 mM, but concentrations are very low in soils near roots and change continuously, varying from 0.1 to 1.0 mM [2]. Thus, most plants will face low K^+^ stress during growth. Most plants can resist low K^+^ stress, mainly because they have established a strategy to acclimate to this stress. Most plants have both high-affinity and low-affinity K^+^ transport systems to sense the K^+^ concentrations in the soil, and the high-affinity system functions when the K^+^ concentrations are below 0.2 mM. The low-affinity system operates at K^+^ concentrations above 0.5 mM; thus, these two transport systems are vital to help plants survive under LK stress [3,4].

Alligator weed (*Alternanthera philoxeroides*) is a dicotyledonous perennial herb that grows worldwide due to its high adaptability to harsh environments. Gao et al. reported that this adaptability is predominantly attributed to genome-wide DNA methylation and epigenetic regulation [5]. This plant was found to have a great ability to accumulate potassium, and three K^+^ transporter (ApKUP1, ApKUP2 and ApKUP3) expression levels were up-regulated by low potassium, abscisic acid (ABA) and polyethylene glycol (PEG) treatments [6]. Comprehensive transcriptome analysis results suggest that 121 transcription factors, 108 kinases, 136 transporters and 178 genes were changed in response to LK stress in alligator weed roots [7].

Proteomics is considered one of the most robust methods; it presents an effective approach to pursue a systems-based perspective of how proteins change and thus how organisms adapt to various abiotic environments [8]. In recent years, gel-free quantitative proteomic methods (e.g., TMT and iTRAQ) have been used as precise and sensitive multiplexed peptide/protein quantification techniques with mass spectrometry [9]. Zeng et al. reported that 129 proteins were related to low K tolerance and were mainly involved in defense, transcription, signal transduction, energy, and protein synthesis in barley [10]. In cotton seedlings xylem sap, 258 peptides were qualitatively identified, of which, 90.31% were secreted proteins. LK significantly decreased the expression of most environmental-stress-related proteins [11]. The proteomics results suggest that most of the differentially abundant proteins were related to jasmonic acid (JA) synthesis, suggesting the importance of JA in the potassium deficiency response in wheat and rice [12]. Physiological and quantitative proteomic analyses reveal the proteins responsible for energy metabolism, signal sensing and transduction, and protein degradation that played crucial roles in alligator weed roots under LK stress [13]. Root-to-shoot translocation and shoot homeostasis of potassium determine the nutrient balance, growth, and stress tolerance of vascular plants. However, the proteomic profile of alligator weed stems under low potassium stress has not yet been studied. In this study, we used a quantitative proteomic analysis method to investigate the protein expression changes and metabolic process networks in alligator weed stems after LK stress. These results will provide critical insights into the potassium tolerance mechanisms of alligator weed.

## 2. Results

### 2.1. Effect of LK Stress on the Physiology of Alligator Weed Stems

**Figure 1:**
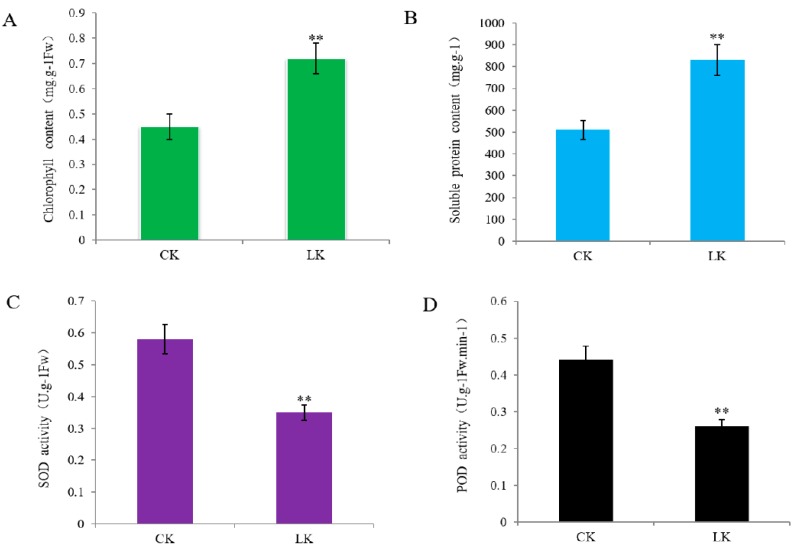
Stem physiology performance were listed. Note (**A**) measurements the total chlorophyll content; (**B**) measurements of soluble protein content; (**C**) measurements of superoxide dismutase (SOD) activity; (**D**) measurements of peroxidase (POD) activity. ** mean that data was significantly different among samples in the same treatment (*p* ≤ 0.05) using the SPSS statistical software. Bars represent the mean ± SE (*n* = 5).

The exposure of alligator weed seedlings to LK stress resulted in various physiological changes in the stem after 15 days of treatment. To determine the physiological responses of the stems after treatment, the chlorophyll and soluble protein content and SOD and POD activity were measured. First, the total chlorophyll content increased by 20% after treatment (Figure 1A), the soluble protein content showed greatly increased (Figure 1B), superoxide dismutase (SOD) and peroxidase (POD) were vitally protective enzymes in plant cells during stress. In the present study, the activity of SOD and POD were both significantly decreased after 15 days of treatment (Figure 1C,D).

### 2.2. Protein Responses to LK Stress Revealed by the Proteomic Analysis

We employed TMT and LC-MS/MS to characterize the proteomic profiles of the stems. The proteome data showed that LK dramatically changed the protein abundance in the stem. The six samples that were analyzed included 3 replicates each of CK and LK stems. A change over 1.2-fold or a cut-off of less than 0.83-fold were considered statistically significant. A total of 296 proteins were altered in expression were regarded as DAPs. Among them, 152 proteins were up-regulated and 144 proteins were down-regulated; their information is shown (Table S1). Among them, 27 DAPs related to transport process were listed (Table 1).

### 2.3. GO and KEGG Analysis of DAPs

**Figure 2:**
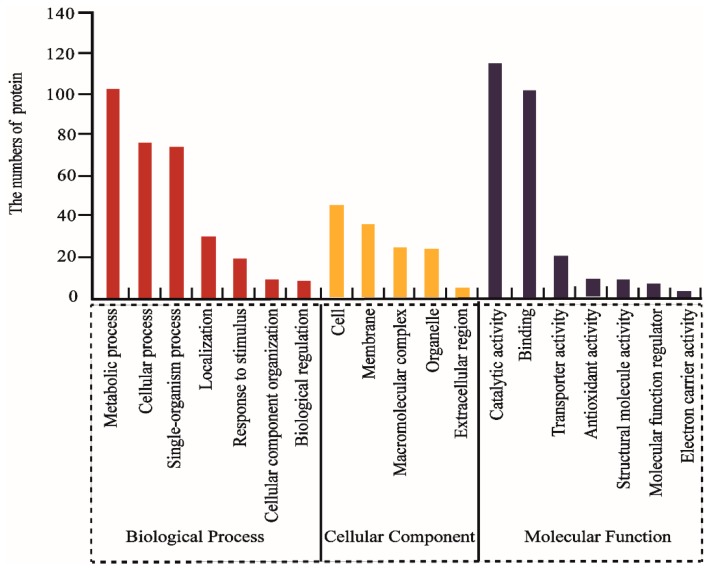
Functional classification of differentially abundant proteins.

**Figure 3:**
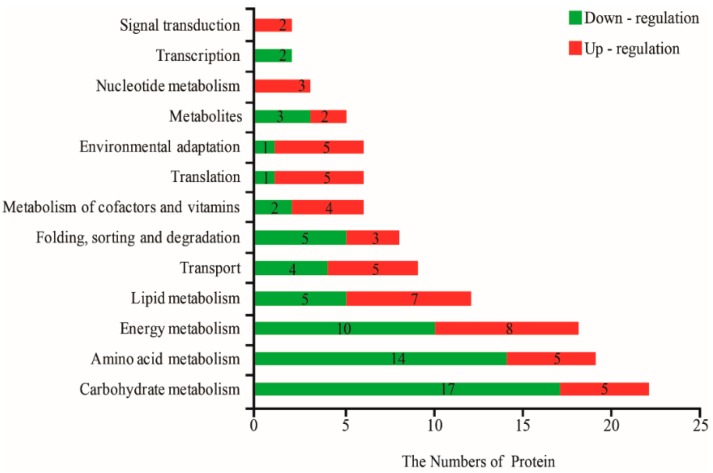
Differentially abundant proteins by KEGG analysis. Note number mean protein number.

All DAPs were annotated and classified according to biological process (BP), molecular function (MF), and cellular component (CC) according to the GO database. The primary categories in BP were metabolic processes, cellular processes, and single-organism processes; the prominent MF categories were catalytic activity, binding, and transporter activity; and the most abundant categories in CC were cell, membrane, and macromolecular complex (Figure 2). Next, the biological metabolic pathways related to 119 DAPs were investigated using KEGG analysis. The results suggested that most represented DAPs were associated with carbohydrate metabolism (20.1%), energy metabolism (15.1%) and amino acid metabolism (15.1%), while the fourth and fifth category was lipid metabolism and transport; every group included 12 to 9 DAPs (Figure 3).

### 2.4. Subcellular Location and Domain Analysis of DAPs

**Figure 4:**
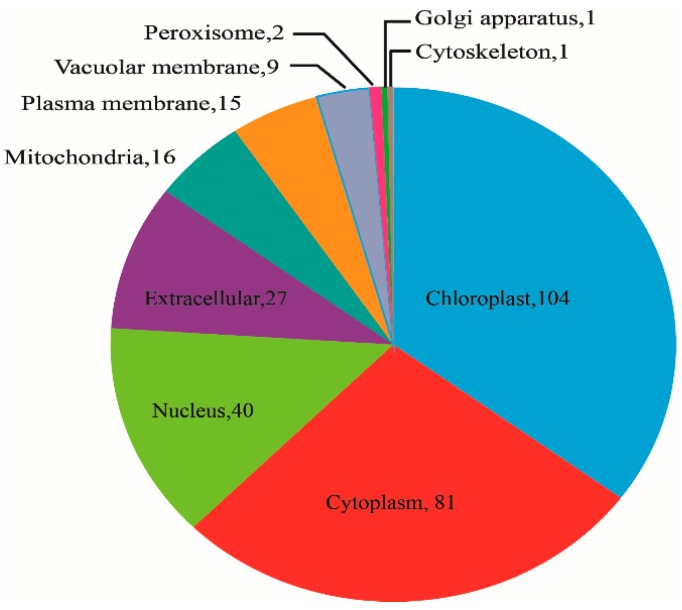
Subcellular locations analysis of differentially abundant proteins. Note number mean protein number in this group.

**Figure 5:**
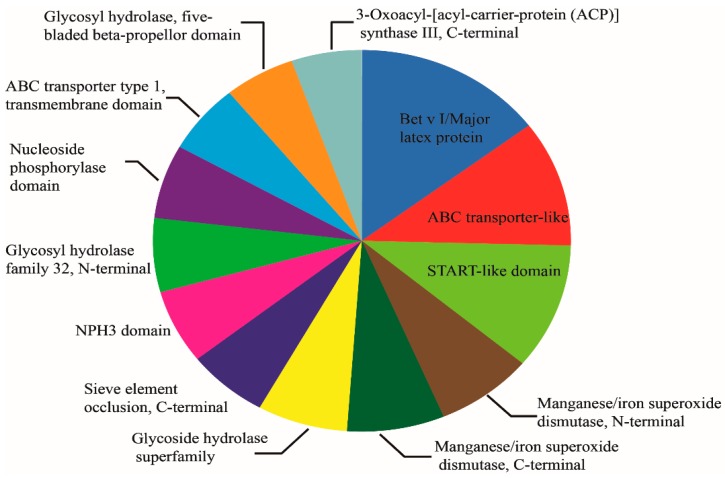
Protein domain analysis of differentially abundant proteins.

The subcellular locations of 296 identified DAPs identified were predicted by Target P1.1 software. The result suggested that 104 proteins showed chloroplastic localization, of which 53 were increased, 81 proteins showed cytoplasmic localization, of which 38 proteins were increased, and 40 showed nuclear localization, of which 25 were increased. These were the top three groups. Localization in the peroxisome, golgi apparatus and cytoskeleton were the lowest, with protein numbers of 2, 1, and 1, respectively (Figure 4). With the protein domain analysis, the top three protein groups were Bet v I/Major latex proteins, ABC transporter-like proteins and START-like domain proteins, and they all mapped 7 proteins. Among them, 7 ABC transporter-like proteins were all up-regulated, and 6 manganese/iron superoxide dismutases (3 N-terminal, 3 C-terminal) were all down-regulated (Figure 5).

### 2.5. Interaction Network Analysis of the DAPs

**Figure 6:**
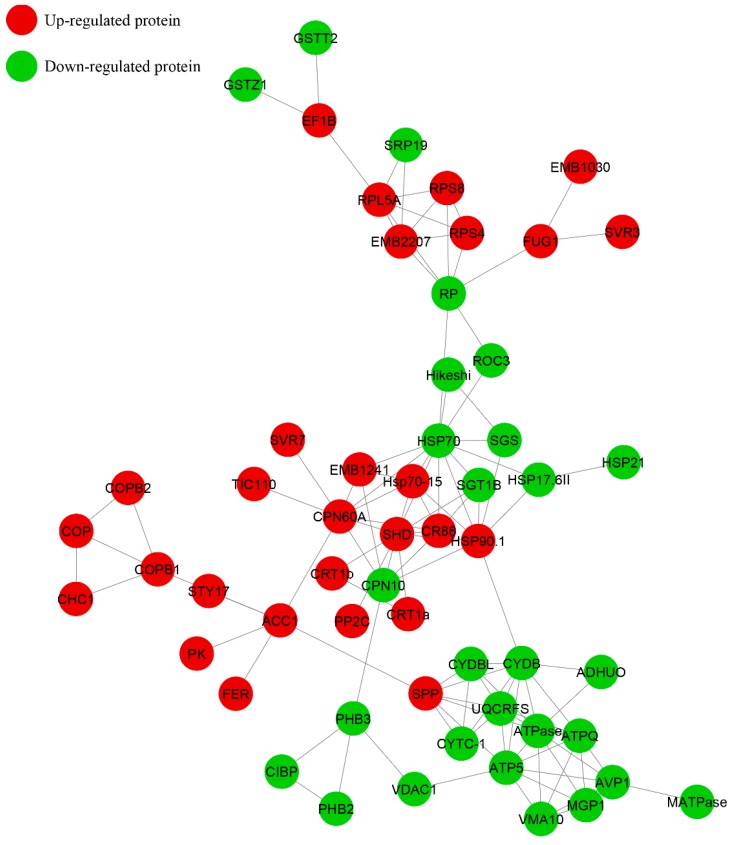
Functional correlation network of differentially abundance protein. Note: RPS8, 30S ribosomal protein S8; EMB2207, 60S ribosomal protein L3; ATP5, ATP synthase subunit O; ATPQ, ATP synthase subunit delta; RPS4, 30S ribosomal protein S4; COPB1, Coatomer subunit beta-1; COPB2, Coatomer subunit beta’-2; COP, Coatomer subunit gamma-2; PHB3, Prohibitin-3; PHB2, Prohibitin-2; RPL5A, 60S ribosomal protein L5; ATPase, ATP synthase subunit delta; CPN60A, Chaperonin 60 subunit alpha 1; CPN10, 10 kDa chaperonin; HSP90.1, Heat shock protein 83; SGT1B, Protein SGT1; SHD, Endoplasmin; CYDB, Cytochrome b-c1 complex subunit 7-2; UQCRFS, Cytochrome b-c1 complex subunit Rieske-2; CYTC-1, Cytochrome c; CR88, Heat shock protein 90-5; HSP70, Heat shock cognate 70 kDa protein; CYDBL, Cytochrome c oxidase subunit 6a; MGP1, Probable ATP synthase 24 kDa subunit; SPP, Stromal processing peptidase; Hsp70-15, Heat shock 70 kDa protein 15; RP, Aribonucleo protein complex subunit 2; CRT1b, Calreticulin; EMB1241, Uncharacterized protein; CRT1a, Calreticulin; ACC1, Acetyl-CoA carboxylase 1; ROC3, Peptidyl-prolyl cis-trans isomerase; SRP19, Signal recognition particle 19 kDa protein; FER, Receptor-like protein kinase; AVP1, Pyrophosphate-energized vacuolar membrane proton pump; PK, Probable receptor-like protein kinase; STY17, Serine/threonine-protein kinase STY17; CIBP, Uncharacterized protein; VMA10, V-type proton ATPase subunit G; CHC1, Clathrin heavy chain 1; SVR3, Translation factor GUF1; FUG1, Translation initiation factor IF-2; GSTT2, Glutathione S-transferase T2; EF1B, Elongation factor 1-delta; ADHUO, NADH dehydrogenase; HSP17.6II, 17.9 kDa class II heat shock protein; Hikeshi, Uncharacterized protein; TIC110, Protein TIC110; VDAC1, Mitochondrial outer membrane protein porin of 36 kDa; SVR7, Pentatricopeptide repeat-containing protein; GSTZ1, Glutathione S-transferase; HSP21, Small heat shock protein; EMB1030, Alanine—tRNA ligase; SGS, Uncharacterized protein; MATPase, mitochondrial ATP synthase; PP2C, Protein phosphatase 2C.

A total of 56 related protein interaction networks were completed, among which 28 were up-regulated and 28 were down-regulated (Figure 6). Nine interaction proteins belonged to the ribosome network. These proteins included 7 up-regulated proteins, such as the 30S ribosomal protein S8 (RPS8), 60S ribosomal protein L3 (EMB2207), 30S ribosomal protein S4 (RPS4), 60S ribosomal protein L5 (RPL5A), chaperonin 60 (CPN60A), heat shock protein 90 (CR88) and heat shock 70 kDa protein 15 (Hsp70-15), and two down-regulated proteins, such as heat shock cognate 70 (Hsp70) and H/ACA ribonucleo protein complex subunit 2 (RP). Nine belonged to the oxidative phosphorylation network, including seven down-regulated proteins, such as Protein SGT1 (SGT1B), chaperonin (CRT1b), cytochrome b-c1 complex subunit 7-2 (CYDB), ATP synthase subunit O (ATP5), cytochrome b-c1 complex subunit Rieske-2 (UQCRFS), two ATP synthase subunit delta (ATPQ, ATPase), and 2 up-regulated proteins, such as endoplasmin (SHD) and heat shock protein 83 (HSP90.1). Other information about proteins is shown in Table S2.

### 2.6. PRM-MS Quantification of DAPs

The expression levels of 5 DAPs were chosen for quantification by PRM-MS analysis to verify the proteomic results (Table 2). As this assay requires the signature peptide of the target protein to be unique, we only selected proteins with a unique signature peptide sequence for the PRM analysis. In the stem, five DAPs were sieve element occlusion, patellin 3, ATP synthase, NAD(P)H dehydrogenase, and glycine-rich RNA-binding protein 5. In general, the fold changes for these detected proteins were in agreement with the findings of proteomic analysis. Our PRM assay illustrated that the proteomic results were credible for further analysis.

### 2.7. Complementation of the Proteomic Results via qRT-PCR

**Figure 7:**
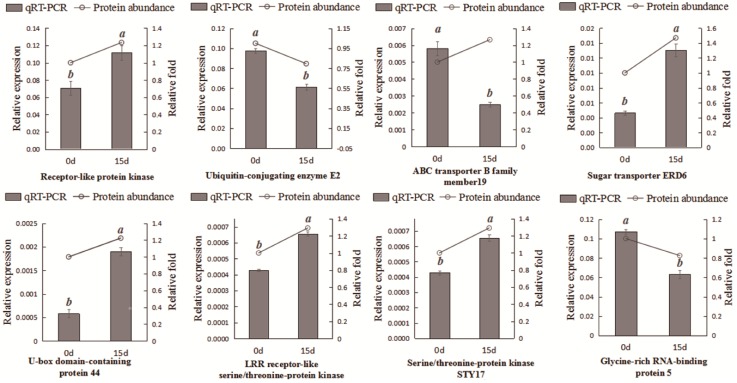
Complementation of proteomic results by qRT-PCR.

A total of eight proteins were randomly selected to complement the accuracy of the proteomics data using quantitative real-time PCR (qRT-PCR). There were 7 gene expression patterns that showed the same tendencies as those for protein expression, including receptor-like protein kinase, ubiquitin-conjugating enzyme E2, sugar transporter ERD6, U-box domain-containing protein 44, LRR receptor-like serine/threonine-protein kinase, serine/threonine-protein kinase STY17, and glycine-rich RNA-binding protein 5. Only one, ABC transporter B family member 19, showed an opposite expression pattern (Figure 7). The primer sequences for eight genes were listed (Table S3). 

## 3. Discussion

### 3.1. LK Affected DAPs Involved in Transport Physiological Process

Membrane proteins fulfill critical functions in the transport of ions and organic molecules, which are an essential part of cellular stress responses. The ABC superfamily proteins mediate the transport across biological membranes not only as ATP-dependent pumps but also as ion channels and channel regulators [14]. Previous reports have suggested that ABC transporters in soybean and tomato are involved in salt stress responses and auxin transport [15,16], Seven ABC transporter proteins increased in expression in our study, suggesting that a high expression of ABC transporters in alligator weed stems are required for potassium transport regulation and LK responses [17,18]. However, further research is required to clarify the details of their function.

Patellin (PATL1) is a membrane trafficking-related protein. In *Arabidopsis*, PATL1 negatively modulates PM Na^+^/H^+^ antiport activity and modulates cellular redox homeostasis during salt stress [19]; in our study, four PATL1s, whose expressions were enhanced, were also identified, showing that they possibly had a vital function in modulating cellular redox homeostasis in alligator weed stems facing LK stress. The sieve element occlusion (SEO) protein is localized to phloem filaments and is required for phloem filament formation and to seal the phloem in wounded tobacco [20,21]; a high SEO expression would protect the stem from damage to maintain the optimum growth of alligator weed. Ca^2+^ wave propagation is channelled through two pore calcium channel protein 1 (TPC1), and the Ca^2+^ wave/TPC1 system likely elicits a systemic molecular response to plant stress tolerance [22]. Moreover, annexin functions as a Ca^2+^-permeable channel in the plasma membrane to mediate the radical-activated plasma membrane Ca^2+^- and K^+^-permeable conductance in root cells [23]. In our study, the TPC1 and annexin proteins were both up-regulated, suggesting that fluxes in the transport of ions from the roots to leaves or post-phloem sugar transport to the root tip help plants rapidly manage stress [24].

One sugar transporter from *Dianthus spiculifolius* affects sugar metabolism and confers osmotic and oxidative stress tolerance in *Arabidopsis* [25]. The overexpression of the MdSUT2.2 gene (sucrose transporter) increased salt tolerance in transgenic apple, and further research suggest that MdSUT2.2 can be phosphorylated by MdCIPK13 and MdCIPK22 to enhance its stability and transport activity [26,27]. In our study, sugar transporter ERD6-like 6 (ERD6) increased in abundance, which may be a vital factor to help alligator weeds improve LK tolerance. Future research is needed to identify the interactions among CIPK proteins. Potassium and nitrogen are essential macronutrients and have a positive impact on crop yield. Previous studies have indicated that the absorption and translocation of K^+^ and NO_3_^−^ are correlated with each other in plants. A lack of NPF7.3/NRT1.5 resulted in K deficiency in shoots under low NO_3_^−^ conditions by affecting xylem loading and root-to-shoot K^+^ translocation through SKOR channel [28]. Further research suggest that NRT1.5 functions as a proton-coupled H^+^/K^+^ antiporter, plays a crucial role in K^+^ translocation from the root to shoot and is also involved in the coordination of K^+^/NO_3_^−^ distribution in plants [29]. Thus, the down-regulation of NRT1/PTR FAMILY 8.3 in our study would decrease nitrate and potassium transport in the root-to-shoot process [30]. These findings provide a basis for the relationship between potassium and nitrogen nutrition in plants.

Syntaxin is a member of the SNARE (soluble *N*-ethylmaleimide-sensitive fusion protein attachment protein receptor) family. Arabidopsis R-SNARE VAMP721 interacts with the inward-rectifying K^+^ channels KAT1 and KC1 and then suppresses the activities of two K^+^ channels [31]. In our study, the decreased abundance of syntaxin-61 could improve KAT1 and KC1 activity to absorb and utilize more K^+^. Recent reports have suggested that the overexpression of two SNARE proteins resulted in high tolerance to drought and salt stress in two kinds of plants [24,32]. Therefore, the SNARE proteins in different plants have multiple functions in regard to abiotic stress.

### 3.2. LK Affected DAPs Related to Carbohydrate and Energy Metabolism

In plants, carbohydrate and energy metabolism, which not only meet the energy demand but also afford many essential cofactors and substrates for other metabolisms and many transport processes, are dependent on the proton motive force that is achieved largely through the H^+^ gradient across membranes afforded by the vacuolar H^+^-ATPase (V-ATPase) [33]. In this study, one V-ATPase was decreased, but its response was different from the root results under LK conditions [13]. In plants, CYTc is also a multi-functional signaling molecule that influences the balance between life and death or triggers programmed cell death [34]. According to the present data, reduced cytochrome c oxidase activity may have reduces cell death in alligator weeds as it does in *Arabidopsis* [35].

Sucrose synthase (Sus) is a key enzyme in sucrose metabolism. One sucrose synthase was observed to be up-regulated, and the same results were found in alligator weed and Arabidopsis under K^+^ deficient conditions [13,36]; a high expression of Sus may play a role in regulating energy metabolism in response to nutrition changes. Uridine-diphospho-(UDP)-glucose 4-epimerase (OsUGE-1) and nitrate reductase (NADH) increased in our study, and recent research has shown that overexpression OsUGEO lines maintain proportionally more galactose than glucose under low N conditions [37]. Nitrate reductase is also necessary under low nitrate stress [38], so we hypothesized that a high abundance of the two proteins could improve K tolerance by increasing N utilization in alligator weed shoots.

Pyruvate kinase (PK) is a glycolysis enzyme that catalyses the conversion of phosphoenolpyruvate (PEP) to pyruvate by transferring a phosphate from PEP to ADP; it has an absolute requirement for K^+^, and a previous study showed that pyruvate kinase has protein kinase activity and plays a role in promoting tumor cell proliferation [39]. Two PKs identified in the present study were up-regulated, possibly having similar functions in plants to promote stem cell proliferation to improve lodging resistance in alligator weeds. Most represented DAPs were associated with carbohydrate and energy metabolism (35.2%) by KEGG analysis (Figure 3), this result was similar to *Arabidopsis* proteomic data [40], meanwhile, nine interaction proteins belonged to the oxidative phosphorylation network (Figure 6), these results supported the change of carbohydrate and energy metabolism were an adjustment mechanism of alligator weed to reduce LK damage.

### 3.3. LK Affected DAPs Related to Photosynthesis

Photosynthesis serves as the major energy source of plants and is directly affected by potassium deficiency. Magnesium chelatase is the first enzyme in the chlorophyll biosynthesis pathway and consists of 3 subunits that include ChlI, ChlD, and ChlH in plants. It is worth mentioning that ChlD and ChlH are related to abscisic acid (ABA) stress in *Arabidopsis*, and over-expression lines of the CHLD gene show more ABA sensitivity than do wild type [41]. ChlH, as an ABA receptor, can be phosphorylated by SnRK2.6 protein kinase [42]. Therefore, the accumulation of these two proteins in our study indicated that ABA pathway-related genes might also play positive roles in enabling plants to adapt LK stress.

Fructose-bisphosphate aldolase is involved in the calvin cycle. Cai et al. reported that the levels of superoxide anions and hydrogen peroxide were increased under low temperature and low-light intensity growth conditions in RNAi tomato seedlings [43]. Because H_2_O_2_ is a vital signal molecule in sensing LK stress [44], a low expression of this enzyme was hypothesized to increase the H_2_O_2_ content and contribute to alligator weed survival in stress.

Three other photosynthesis-related proteins, including one carbonic anhydrase (CA) and two oxygen-evolving enhancer protein 3 (OEE3), were down-regulated. CA plays a crucial role in the CO_2_-concentrating mechanism (CCM), and recent research has shown that OEE is also an excellent antioxidant [45]. Additionally, *hcar* mutants (7-hydroxymethyl chlorophyll a reductase) showed an accelerated cell death phenotype due to excessive accumulation of singlet oxygen in rice and Arabidopsis, but HCAR-overexpressing plants were more tolerant to reactive oxygen species than were the *hcar* mutants [46]. HCAR and ribulose bisphosphate carboxylase were decreased in our study; therefore, it may be assumed that the down-regulation of photosynthesis-related proteins are associated with the LK stress response in the stems. The subcellular locations analysis revealed.

104 proteins were chloroplastic localization (Figure 4), the possible reason was that more proteins synthesized by the leaves were transported to the stems, or the stems cell synthesized more proteins for photosynthesis under LK stress for survival in alligator weed.

### 3.4. LK Affected DAPs Related to Common Stress Responses 

LK stress may disturb cellular redox homeostasis and promote the production of reactive oxygen species (ROS); ROS can be scavenged by plant antioxidant defense systems consisting of a series of enzymes, such as superoxide dismutase (SOD), peroxidases (POD), glutathione-s-transferase (GST) and glutathione peroxidase (GPX). The expression of these enzymes were found to be changed in our proteomic data. Under LK conditions, these proteins are involved in detoxifying ROS to maintain the homeostasis of these molecules in the cytosol. Pattanayak et al. reported that the overexpression of protochlorophyllide reductase (PORCx) regulates oxidative stress in *Arabidopsis* and that overexpression reduced the generation of ^1^O_2_ to reduce plasma membrane damage [47]. One PORCx was increased in our study. Thus, it may be assumed that this protein was helpful in facing LK stress responses. In summary, the expression changes of these proteins implied that the antioxidative defense system was provoked in the stems to protect alligator weed. Major latex protein (MLP)-like protein, as a positive regulator of downstream signaling, mainly responds to defense or abiotic stimuli [48]. In our study, two MLP-like proteins were remarkably increased, one was decreased. Previous research has suggested that the over-expression of MLP protein enhances the salt and drought tolerance of Arabidopsis [49]; however, the specific biological function of MLP related to LK was unknown, so it may be a novel LK-stress-responsive protein in alligator weed plants. In the current study, three heat shock proteins were found to be significantly up-regulated, while three were down-regulated. The expression differences imply that the gene family members probably have diverse functions to cope with various stresses [50].

Meanwhile, one chaperonin 60 showed enhanced expression, and the Oscpn60α1 mutant had a pale-green phenotype at the seedling stage. Further analysis indicated that the level of the rubisco large subunit (rbcL) was severely reduced in the mutant, so OsCpn60α1 is required for the folding of rbcL [51]. Therefore, a high expression of chaperonin 60 may maintain the correct protein folding under LK stress in the stem. Universal stress-related proteins are also redox-dependent chaperones, and a high expression of these proteins enhances plant tolerance to heat shock and oxidative stress in *Arabidopsis* [52]. Three stress-related proteins were found to be significantly down-regulated. In addition, other stress proteins, such as remorins, phenylalanine ammonialyase (PAL) and cellulose synthase, were all up-regulated. Taken together, these results suggest that the stress response proteins all play significant roles in improving the ability of alligator weed to cope with abiotic stress.

### 3.5. Comparative Analysis of Low-K^+^ Responses between Alligator Weed and Arabidopsis

Phospholipids play crucial roles in regulating development and signal transduction in higher plants, protein phosphatase 2C (PP2C) physically interacts with CBL-CIPK and can activate the AKT1 channel in *Arabidopsis* [53]. AtPP2A plays vital roles for root auxin transport, gravity response, and lateral root development [54]. In our study, two protein phosphatase 2C and one phospholipase A were found to be up-regulated, this is consistent with the proteomic results in *Arabidopsis* seedlings after LK treatment [40]. These results indicated that high expression PP2C and PP2A maybe induce root growth and absorb more K^+^ during low potassium conditions, Therefore, it is clear that phospholipids signal regulation pathway is conservative in plant. Meanwhile, the calcium signaling pathway is vital in phosphorylation signal transduction responses under stress conditions. Calreticulin (CRT) is a multifunctional protein that participates in plant growth and development as an important molecular partner on the endoplasmic network. CRT mutant exhibit more sensitivity to water stress in *Arabidopsis* [55]. In Arabidopsis CRT1 and CRT2 are critical components in the accumulation of VESICLE-ASSOCIATED MEMBRANE PROTEIN 721 (VAMP721) and VAMP722 during ER stress responses [56]. Arabidopsis VAMP721 could assemble with SYP121 to drive membrane fusion and bind to the KAT1 K^+^ channel to control channel-gating [57]. In our data, two calreticulin (CRT) increased abundance but this protein was not reported in proteomic analysis under LK stress before, so this result raised CRT maybe maintain intracellular calcium homeostasis or interacts with VAMP721 to improve K^+^ channels activity to increase LK tolerance in alligator weed.

The receptor-like protein kinases (RLKs) are involved in plant growth and development, hormonal responses, cellular differentiation, stress responses and pathogen recognition [58]. The heterologous overexpression of one serine/threonine-protein kinase (STK) from *Bambusa balcooa* in transgenic tobacco induce higher cellulose synthesis and high cellulose deposition in the xylem fibres, increase a greater number of xylary fibres and enhance mechanical strength in transgenic tobacco [59]. In our study, the expression of one STK was enhanced in the stem. At the same time, cellulose synthase was also increased, so we hypothesize that high STK expression can maintain the stem mechanical strength under LK stress by improving cellulose synthase activity. Previous research suggested RLKs can recognized CLE (CLAVATA3/Endosperm surrounding region-related) to form CLE-RLK module in order to convey extracellular and intracellular signaling in long distance [60], in our study, three RLKs increased abundance, maybe these protein kinase can interaction with CLE peptides to re-establish new optimal growth state under LK stress, this result could partly explain the high LK tolerance of alligator weeds because of good root to shoot signaling.

Some protein involved in protein synthesis was founded to be increased abundance in the present data, including two 30S ribosomal proteins, two 60S ribosomal proteins, three translation initiation factors and one translation factor. The overexpression of elongation factors in plants allow them to be more tolerant to high temperature stress [61], so it has been noticed that the accumulation of elongation factors could play a role in splicing, polyadenylation, mRNA stabilization and localization, and translation [62]. The expression of four RNA-binding protein were significantly increased. Nine interaction proteins belonged to the ribosome network after analysis (Figure 6), these results showed that protein synthesis and post-transcriptional regulation processes were improved in stems after LK treatment for 15 days compare to *Arabidopsis*. RBPs that consist of both an RNA-recognition motif (RRM) and a glycine-rich region termed GRPs [63]. Kim et al. reported GRPs are functionally conserved during the cold adaptation process in rice and *Arabidopsis* as RNA chaperones [64]. Glycine-rich RNA-binding protein (GRP) expression has been shown to be regulated by different abiotic stresses, including low temperature, water deficit and high salinity stress, and over-expressing ZjGRP from *Zoysia japonica* weaken salinity tolerance in *Arabidopsis*, affecting ion transportation, osmosis, and antioxidation [65]. The expression of GRP5 was reduced, and the result was confirmed by qRT-PCR and PRM analysis. Therefore, it can be hypothesized that the low expression of this protein may play a positive role in improving LK tolerance in alligator weeds. It was different to *Arabidopsis*.

Ubiquitination is a post-translational modification of proteins process consisting ubiquitin activating enzyme (E1), ubiquitin-conjugating enzyme (E2), and ubiquitin ligase enzyme (E3), which regulates a wide range of biological processes, including adaptation to drought, salinity, cold, and nutrient deprivation [66], which the overexpression of the soybean ubiquitin-conjugating enzyme gene GmUBC2 in *Arabidopsis* enhance its drought and salt tolerance by modulating abiotic stress-responsive gene expression [67]. It was worth noting that three E2 were decreased, which was similar to the proteomic results in *Arabidopsis* under LK stress for 7 days [40], and the role of E2s may be similar to those of AtUBC32, AtUBC33, and AtUBC34, because the suppression of three E2 expression level result in increased abscisic acid-mediated stomata closure and tolerance to drought stress [68]. Therefore, the three E2s in the stems play negative roles under LK stress, and this result can also provide clues for subsequent in-depth research.

**Figure 8:**
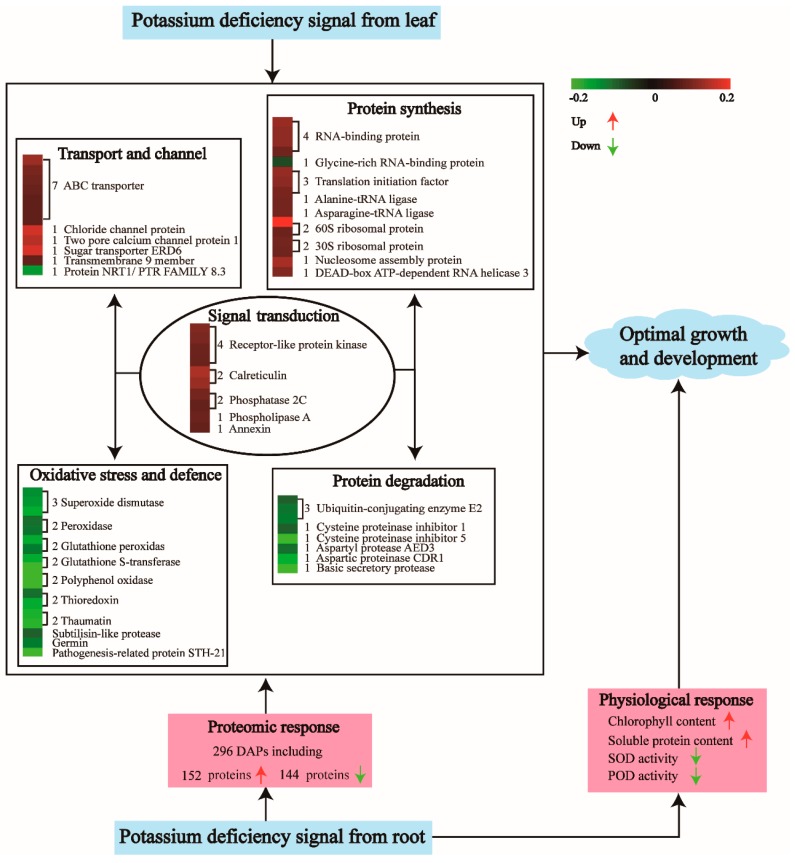
Model showed the physiological and proteomic responses of alligator weed stem under potassium deficiency (LK) stress.

In conclusion, much new information about the protein profile in alligator weed stems was obtained with respect to LK stress (Table S4), the physiological analysis and expression of these proteins were a clear response to potassium deficiency in stem (Figure 8). These proteins worked together to establish a new steady-state balance of metabolic processes to enable alligator weed to grow normally after enduring LK stress for 15 days.

## 4. Materials and Methods

### 4.1. Physiological Experiments of Alternanthera philoxeroides Stem

Naturally grown *Alternanthera philoxeroides* shoots were collected from a test field of Sichuan Agricultural University (Chengdu, China) and cultured hydroponically in a growth chamber for 10 day to induce root growth. The greenhouse was maintained under a 16 h/8 h day/night light cycle and a 28 °C/25 °C day/night temperature cycle.

The nutrient solution was refreshed every 2 day. The nutrient solution was prepared as described before [7]. The alligator weed plants had grown strong roots after 10 day of culture. Half of the plants were then transferred to a low potassium nutrient solution (lacking K_2_SO_4_) as the LK sample. The other half continued to grow in the solution with optimum K concentration as the CK sample. Then, LK and CK alligator weed stem samples were collected after 15 day of LK treatment for the physiological and molecular measurements. The chlorophyll and soluble protein content and SOD and POD activity were measured. The total chlorophyll and soluble protein content and SOD and POD activity were measured as described previously [15,50].

### 4.2. Protein Extraction

Six stem samples were ground in liquid nitrogen to cell powder and then transferred to a 5-mL centrifuge tube. Then, four volumes of lysis buffer (8 M urea, 1% Triton-100, 10 mM dithiothreitol, and 1% protease inhibitor cocktail) were added to the cell powder, followed by sonication three times on ice using a high intensity ultrasonic processor. The remaining debris was removed by centrifugation at 20,000× *g* at 4 °C for 10 min, and finally, the protein was precipitated with cold 20% TCA for 2 h at −20 °C. After centrifugation at 12,000× *g* at 4 °C for 10 min, the supernatant was discarded, the remaining precipitate was washed with cold acetone three times, the protein was redissolved in 8 M urea and the protein concentration was determined with a BCA kit according to the manufacturer’s instructions.

### 4.3. Trypsin Digestion and TMT Labelling

For digestion, the protein solution was reduced with 5 mM dithiothreitol for 30 min at 56 °C and alkylated with 11 mM iodoacetamide for 15 min at room temperature in the dark. The protein sample was then diluted by adding 100 mM TEAB to obtain a urea concentration less than 2 M. Finally, trypsin was added at a 1:50 trypsin-to-protein mass ratio for the first digestion overnight and a 1:100 trypsin-to-protein mass ratio for a second 4 h digestion. After trypsin digestion, the peptides were desalted using a Strata X C18 SPE column (Phenomenex) and vacuum dried. The peptide were reconstituted in 0.5 M TEAB and processed according to the manufacturer’s protocol for the TMT kit. Briefly, one unit of TMT reagent was thawed and reconstituted in acetonitrile. The peptide mixtures were then incubated for 2 h at room temperature, pooled, desalted and dried by vacuum centrifugation.

### 4.4. HPLC Fractionation

The tryptic peptides were fractionated by high pH reverse-phase HPLC using an Agilent 300Extend C18 column (5 μm particles, 4.6 mm ID, and 250 mm length). Briefly, the peptides were first separated with a gradient of 8% to 32% acetonitrile (pH 9.0) over 60 min into 60 fractions. Then, the peptides were combined into 18 fractions and dried by vacuum centrifugation.

### 4.5. LC-MS/MS Analysis

The tryptic peptides were dissolved in 0.1% formic acid (solvent A) and directly loaded onto a homemade reversed-phase analytical column. The gradient was comprised of an increase from 7% to 25% solvent B (0.1% formic acid in 98% acetonitrile) over 38 min and 25% to 40% over 14 min and then rose to to 80% for 4 min, followed by holding at 80% for the last 4 min, all at a constant flow rate of 700 nL/min on an EASY-nLC 1000 UPLC system. The peptides were subjected to an NSI source followed by tandem mass spectrometry (MS/MS) by using a Q Exactive^TM^ Plus (Thermo, Waltham, MA, USA) coupled online to the UPLC. The electrospray voltage applied was 2.0 kV. The *m*/*z* scan range was 350 to 1000 for the full scan, and the intact peptides were detected in the Orbitrap at a resolution of 70,000. The peptides were then selected for MS/MS using the NCE setting at 27, and the fragments were detected in the Orbitrap at a resolution of 17,500. A data-independent procedure that alternated between one MS scan followed by 20 MS/MS scans was performed. The automatic gain control (AGC) was set at 3E6 for the full MS and 1E5 for MS/MS. The maximum IT was set at 20 s for the full MS and auto for MS/MS. The isolation window for MS/MS was set at 2.0 *m*/*z*.

### 4.6. Database Search

The resulting MS/MS data were processed using the Maxquant search engine (v.1.5.2.8). The tandem mass spectra were searched against alligator weed transcription data. Trypsin/P was specified as a cleavage enzyme allowing up to two missing cleavages. The mass tolerance for the precursor ions was set as 20 ppm in the first search and 5 ppm in the main search, and the mass tolerance for the fragmented ions was set as 0.02 Da. Carbamidomethyl on Cys was specified as the fixed modification, and oxidation on Met was specified as the variable modifications. FDR was adjusted to <1%, and the minimum score for the peptides was set to >40.

### 4.7. DAPs Functional Analysis

The DAPs were assigned to the NCBI non-redundant (Nr) protein database using the Blast 2GO program to obtain their functional annotation. The Gene Ontology (GO) annotation proteome was derived from the UniProt-GOA database (available online: http://www.ebi.ac.uk/GOA/). The Kyoto Encyclopedia of Genes and Genomes (KEGG) database was used to annotate the protein metabolic pathways.

GO terms or KEGG pathways with *p*-values < 0.05 were regarded as significantly enriched. The DAPs subcellular location prediction was conducted followed by TargetP1.1 (available online: http://www.cbs.dtu.dk/services/TargetP/). Finally, the protein–protein interactions were analyzed for the identified proteins using the STRING v10 database (available online: http://strin g-db.org) to determine their functions and pathways.

### 4.8. Parallel Reaction Monitoring PRM-MS Analysis

The protein expression change obtained using the proteomic analysis were confirmed by a PRM-MS analysis carried out at Jingjie PTM-Biolab Co., Ltd. (Hang Zhou, China). The proteins (60 μg) from the stem sample were prepared, reduced, alkylated, and digested with trypsin following the protocol for the TMT analysis. The obtained peptide mixtures were introduced into the mass spectrometer via a C18 trap column (0.10 × 20 mm; 3 μm) and then via a C18 column (0.15 × 120 mm; 1.9 μm). The raw data obtained were then analyzed using Proteome Discoverer 1.4 (Thermo Fisher Scientific). The FDR was set to 0.01 for the proteins and peptides. Skyline 2.6 software (Download from the MacCoss Lab at the University of Washington) was used for the quantitative data processing and proteomic analysis.

### 4.9. Quantitative Reverse Transcription PCR (qRT-PCR) Analysis

For the qRT-PCR analysis, total RNA was extracted from the stem after 15 days of LK treatment using TRIzol reagent (Invitrogen, Carlsbad, CA, USA). The isolated total RNA was used to generate cDNA with a reverse transcriptase kit (Thermo, Tokyo, Japan). The relative quantification of the candidate genes by qRT-PCR was carried out using a 7500 Real Time PCR System machine (Bio-Ras, Life Technologies, Carlsbad, CA, USA) following the manufacturer’s protocols. The formula 2^−ΔΔ*C*t^ was used to calculate the relative gene expression levels. Actin2/8 expression was used as the internal control, and three replicates were conducted. All data are shown as the mean ± SD (*n* = 3).

### 4.10. Statistical Analysis

For all generated data, at least three biological replicates were performed for the chlorophyll and soluble protein content measurements and SOD and POD activity measurements. The data were subjected to unpaired student’s *t*-tests at levels of *p* ≤ 0.01 and *p* ≤ 0.05. The data are shown as the mean ± SE (*n* = 3). Excel and the SPSS 14.0 statistical software package were used for the statistical analyses of the data. The statistical results are reported as the mean ± SD.

## Figures and Tables

**Table 1:** 27 differentially abundant proteins (DAPs) related to transport process were listed.

Protein Accession Number	Protein Annotation	Protein Score	Percentage of Protein Sequence Coverage %	Peptides Count	Number of Unique Peptides	Fold Change	*p*-Value
Gene.10143	ABC transporter F family member 5	52.24	8.1	5	5	1.34	0.0425
Gene.15326	ABC transporter B family member 19	91.58	10	11	8	1.27	0.00104
Gene.11683	ABC transporter F family member 4	45.75	6	3	3	1.25	0.0222
Gene.22137	ABC transporter B family member 1	72.34	9.9	11	3	1.23	0.00932
Gene.37843	ABC transporter C family member 10	7.88	3.8	1	1	1.21	0.0478
Gene.39686	ABC transporter D family member 2	30.70	3.1	2	2	1.21	0.0289
Gene.14866	ABC transporter B family member 2	107.13	11.8	12	10	1.21	0.0108
Gene.22512	Protein sieve element occlusion	30.17	5.9	4	4	1.29	0.00538
Gene.1036	Protein sieve element occlusion	19.51	26.9	3	3	1.29	0.00456
Gene.16899	Protein sieve element occlusion	323.31	35.3	31	29	1.27	0.000702
Gene.29767	Patellin-3	286.67	34.9	24	10	1.39	0.0226
Gene.42322	Patellin-3	25.04	25.7	4	4	1.34	0.0464
Gene.25481	Patellin-4	7.54	2.1	1	1	1.29	0.0105
Gene.42892	Patellin-5	12.17	8.9	3	2	1.85	0.0142
Gene.22040	Sugar transporter ERD6-like 6	46.34	3.8	1	1	1.47	0.000758
Gene.19069	Chloride channel protein CLC-b	13.79	2.4	2	2	1.46	0.042
Gene.44796	Two pore calcium channel protein 1	80.37	7.5	5	5	1.39	0.0196
Gene.41564	Vesicle-associated membrane protein	32.77	41.5	4	2	1.26	0.0172
Gene.23148	Transmembrane 9 superfamily member	14.87	8.8	6	2	1.21	0.0113
Gene.36797	Kinesin-like protein KIN-UA	9.03	1.8	1	1	1.21	0.0246
Gene.1641	Vacuolar membrane proton pump	25.00	12.9	9	1	0.81	0.0441
Gene.51508	Chloroplastic lipocalin	41.09	17.7	5	5	0.78	0.784
Gene.38083	Stem-specific protein TSJT1	48.56	18.5	4	4	0.78	0.0021
Gene.14846	Protein NRT1/PTR FAMILY 8.3	34.84	6.5	4	2	0.74	0.0466
Gene.1247	Syntaxin-61	6.42	4.5	1	1	0.78	0.0021
Gene.1250	Syntaxin-61	6.32	3.7	1	1	0.67	0.0248
Gene.11391	V-type proton ATPase subunit G	155.18	62.7	8	8	0.7	0.0448

**Table 2:** Confirmation of DAPs s in proteomic analysis using PRM analysis.

Description	Change in TMT	*p*-Value in TMT	Change in RPM	*p*-Value in RPM
Sieve element occlusion	1.27	0.000702	1.28	0.005
Patellin-3	1.39	0.0226	1.49	0.03
ATP synthase	0.83	0.00086	0.74	0.0015
NAD(P)H dehydrogenase	0.81	0.00398	0.88	0.01
Glycine-rich RNA-binding protein 5	0.83	0.0457	0.67	0.002

## Supplementary Materials

The following are available online at http://www.mdpi.com/1422-0067/20/1/221/s1.
